# Towards malaria elimination: a reflection about digital notification modules to improve malaria cases notification speed and follow-up in the Brazilian Amazon region

**DOI:** 10.1186/s12936-024-04971-6

**Published:** 2024-05-23

**Authors:** Klauss Kleydmann Sabino Garcia, Sheila Rodrigues Rodovalho, André M. Siqueira

**Affiliations:** 1 Center for Tropical Medicine, University of Brasília, Brasília, Brazil; 2 Faculty of Health Sciences, University of Brasília, Brasília, Brazil; 3 Pan American Health Organization (PAHO), Brasilia, Brazil; 4 Fiocruz, Instituto Nacional de Infectologia Evandro Chagas, Rio de Janeiro, Brazil

**Keywords:** Brazil, Disease notification, Epidemiology, Health information systems, Malaria, Mobile applications

## Abstract

**Background:**

Health information systems (HIS) are a pivotal element in epidemiological surveillance. In Brazil, malaria persists as a public health challenge, with 99% of its occurrences concentrated in the Amazon region, where cases are reported through the HIS Sivep-Malaria. Recent technological advancements indicate that case notifications can be expedited through more efficient systems with broader coverage. The objective of this study is to analyse opportunities for notification within Sivep-Malaria and explore the implementation of mobile electronic devices and applications to enhance the performance of malaria case notifications and use.

**Methods:**

This descriptive study analyses data on malaria-positive cases in the Brazilian Amazon from 2004 to 2022. Malaria Epidemiological Surveillance System (Sivep-Malaria) data were used. The Brazilian Amazon region area is approximately 5 million km^2^ across nine different states in Brazil. Data entry opportunities were assessed by considering the time difference between the 'date of data entry' and the 'date of notification.' Descriptive statistics, including analyses of means and medians, were conducted across the entire Amazon region, and for indigenous population villages and gold mining areas.

**Results:**

Between 2004 and 2022, 6,176,878 new malaria cases were recorded in Brazil. The average data entry opportunity throughout the period was 17.9 days, with a median of 8 days. The most frequently occurring value was 1 day, and 99% of all notifications were entered within 138 days, with 75.0% entered within 20 days after notification. The states with the poorest data entry opportunities were Roraima and Tocantins, with averages of 31.3 and 31.0 days, respectively. For indigenous population villages and gold mining areas, the median data entry opportunities were 23 and 15 days, respectively.

**Conclusions:**

In malaria elimination, where surveillance is a primary strategy for evaluating each reported case, reducing notification time, enhancing data quality and being able to follow-up cases through computerized reports offer significant benefits for cases investigation. Technological improvements in Sivep-Malaria could yield substantial benefits for malaria control in Brazil, aiding the country in achieving disease elimination and fulfilling the Sustainable Development Goals.

**Supplementary Information:**

The online version contains supplementary material available at 10.1186/s12936-024-04971-6.

## Background

### Health information systems

Health Information Systems (HIS) are instruments for data collection and surveillance [1]. Their primary objective is to provide information for the analysis and better understanding of significant health issues within the population, supporting decision-making at the municipal, state, and federal levels [2].

In Brazil, the development, expansion, and use of HIS at the national level have accompanied the definition, regulation, and organization of the Unified Health System (SUS—*Sistema Único de Saúde*) in healthcare networks, enhanced by the rapid development and incorporation of information and communication technologies that took place in the country from the 1990s onwards [2].

A case notification is the communication of the occurrence of a disease or health event, which must be reported to health authorities. In Brazil, systematic disease and health events notification started after the Smallpox Eradication Campaign in 1969 [3]. Thus, reporting an illness or event is one of the primary strategies for monitoring public health, decision-making, and timely intervention use [4].

### Malaria case notification

Malaria is a compulsory notifiable disease in Brazil, and it is considered a significant public health problem worldwide, being one of the most impactful diseases in terms of morbidity and mortality in populations situated in tropical and subtropical regions. In 2022, 249 million new cases and 608,000 deaths from malaria were reported globally [5].

In Brazil, malaria transmission is predominant in the Amazon Region, accounting for approximately 99% of the country's cases [6]. In 2022, 131,224 cases were recorded, a national reduction of 6.6% compared to 2021 (140,488). Also, 98.3% of notified cases were infected in Brazilian territory and 84.2% of them were caused by *Plasmodium vivax* [7].

The National Malaria Control and Prevention Programme (NMCP) of the Ministry of Health of Brazil currently works to eliminate autochthonous malaria cases until 2035 and malaria deaths until 2030. However, the country still faces challenges reaching those goals, such as controlling malaria infection in indigenous populations villages, and in gold mining areas, especially infections caused by *Plasmodium falciparum* [6].

Since 2003, the NMCP has implemented the Malaria Epidemiological Surveillance Information System (Sivep-Malaria) as its primary surveillance tool. This system has contributed to malaria control in the Brazilian Legal Amazon [8]. The notification of malaria cases is mandatory in the country and must be done in Sivep-Malaria when notification is in the Amazon region [9]. The NMCP considers all states in the northern region of Brazil combined with the states of Maranhão and Mato Grosso as the Amazon region.

The malaria case notification form, updated in August 2020, contains 54 fields, not all of which are mandatory, divided into notification data (#2–11), patient data (#12–35), probable location of infection (#36–41), examination data (#42–51), and treatment (#52–54) (Additional File 1). Filling out the notification form is mainly divided between the reporting agent (up to field 41) and the professional performing the laboratory diagnosis (remaining fields), predominantly the microscopist.

The malaria case notification is initiated by completing the printed notification form of Sivep-Malaria locally by the healthcare professional, who submits the physical notification forms to the Municipal Health Departments (MHDs) for data entry. Healthcare professionals mostly conduct case notifications at various levels of care, including hospitals, basic health units, diagnostic centers, and reporting agents who conduct active case detection.

**Fig. 1:**
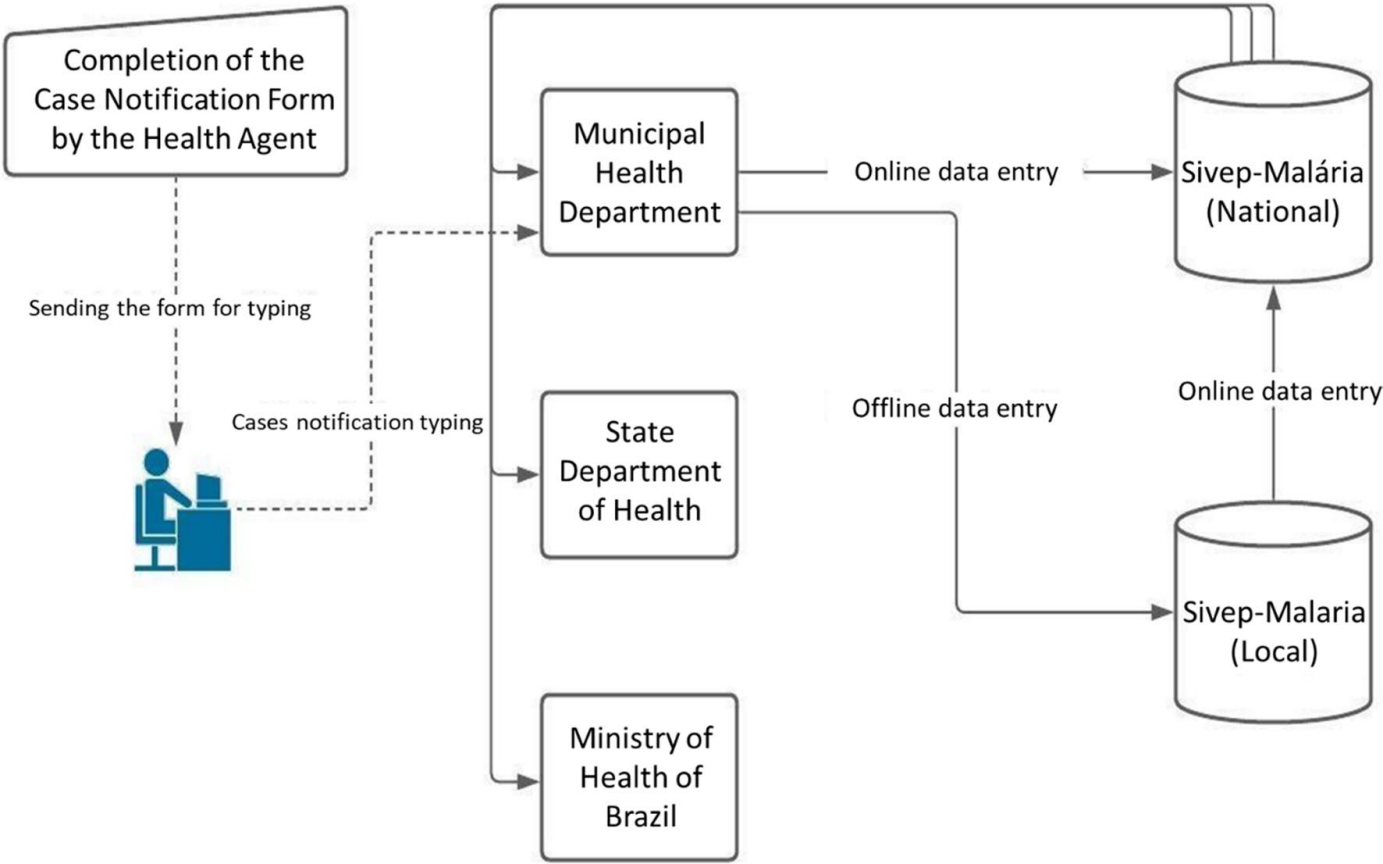
Scheme of malaria case input into Sivep-Malaria. Source: Adapted from Braz et al. [8]

When the local health facility lacks internet access, data entry can be performed in the local system (offline) and saved on digital media for later submission to the national system. When internet access is available, data entry occurs directly in the national system and can be analysed by the three levels of malaria surveillance management (Fig. 1).

### Contemporary computerization

The proliferation of mobile technologies, coupled with advancements in their application to health priorities, has given rise to a new field known as mHealth [10]. Mobile health apps are being tested in various settings to enhance access to health services and information, manage patient care, alleviate drug shortages, and improve clinical diagnosis and treatment adherence, among other benefits [10].

Brazil has around 282 million mobile phones, 90% of which operate on the Android system. Many Brazilians (64%) use mobile applications to seek information, access files, or communicate [11]. Thus, discussing the possibility of using mobile device applications for malaria case notification presents an opportunity to streamline malaria epidemiological surveillance.

Therefore, this article aims to analyse the opportunities for notification within Sivep-Malaria, aiming to reflect on the implementation of mobile electronic devices and applications to improve malaria cases notification performance and use.

## Methods

### Study design

This is a descriptive study used data from malaria cases reported in the Brazilian Amazon region from 2004 to 2022. The Ministry of Health of Brazil provided the data through the Fala.BR platform (Integrated Platform for Ombudsman and Access to Information, Protocol #25072.032117/2023-34), and data were updated in June 2023.

### Study site

**Fig. 2:**
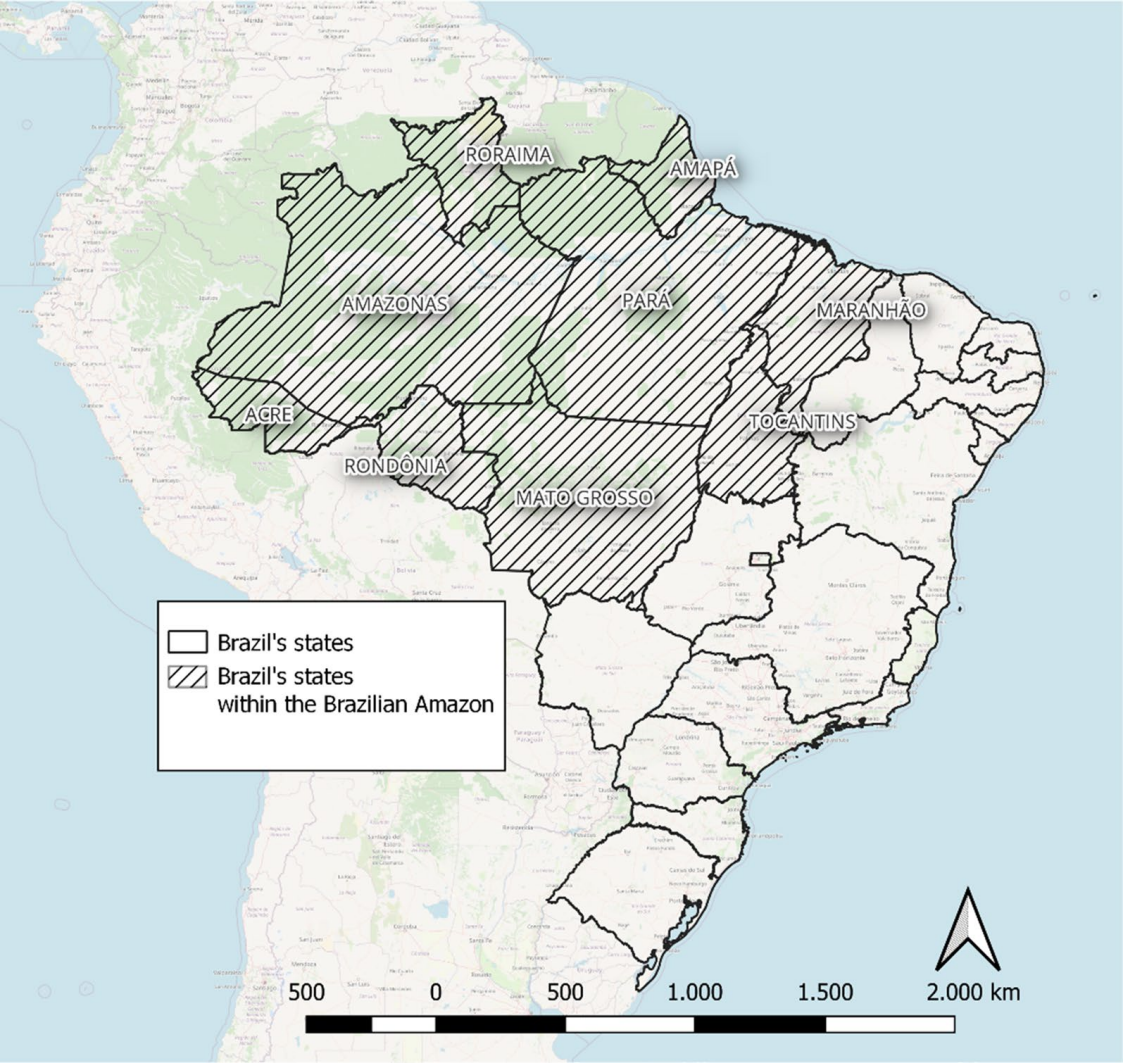
The Brazilian Amazon region

The Brazilian Amazon region has an approximate area of 5,015,067.86 km^2^ and encompasses the states of Acre, Amazonas, Amapá, Mato Grosso, Maranhão, Pará, Roraima, Rondônia, and Tocantins (Fig. 2). The population of these states in 2022 was 28,419,606 inhabitants. The region represents over 60% of Brazil's total landmass and contains the largest contiguous tropical rainforest in the world [12]. Cities in the Amazon region can vary in structure, ranging from metropolises to small villages. Some cities are accessible only by boat, via the Amazon River. Additionally, there are several indigenous population villages and numerous areas with mining activities. These areas can pose challenges for epidemiological surveillance and health assistance [12, 13].

### Statistical analysis

The variables utilized in data analysis were ‘Date of notification’, ‘date of data entry’, ‘location of notification’, “Place of infection” and ‘examination result’ were considered. All notifications of positive malaria cases, including recurrences, were included. Data analysis used descriptive statistics, including means, medians, and interquartile ranges.

The analysis of ‘data entry opportunity’ considered the difference in days between the 'date of data entry' and the 'date of notification'. Records where the date of data entry preceded the date of notification were considered data entry errors and were treated as ‘not available’.

All data treatment and statistical analysis were conducted utilizing R software (version 4.2.3) and Qgis software (version 3.28) for maps production.

### Ethics statement

This study did not require approval from ethics committees, as per Resolution No. 510/2016 of the Brazilian National Health Council.

## Results

**Fig. 3:**
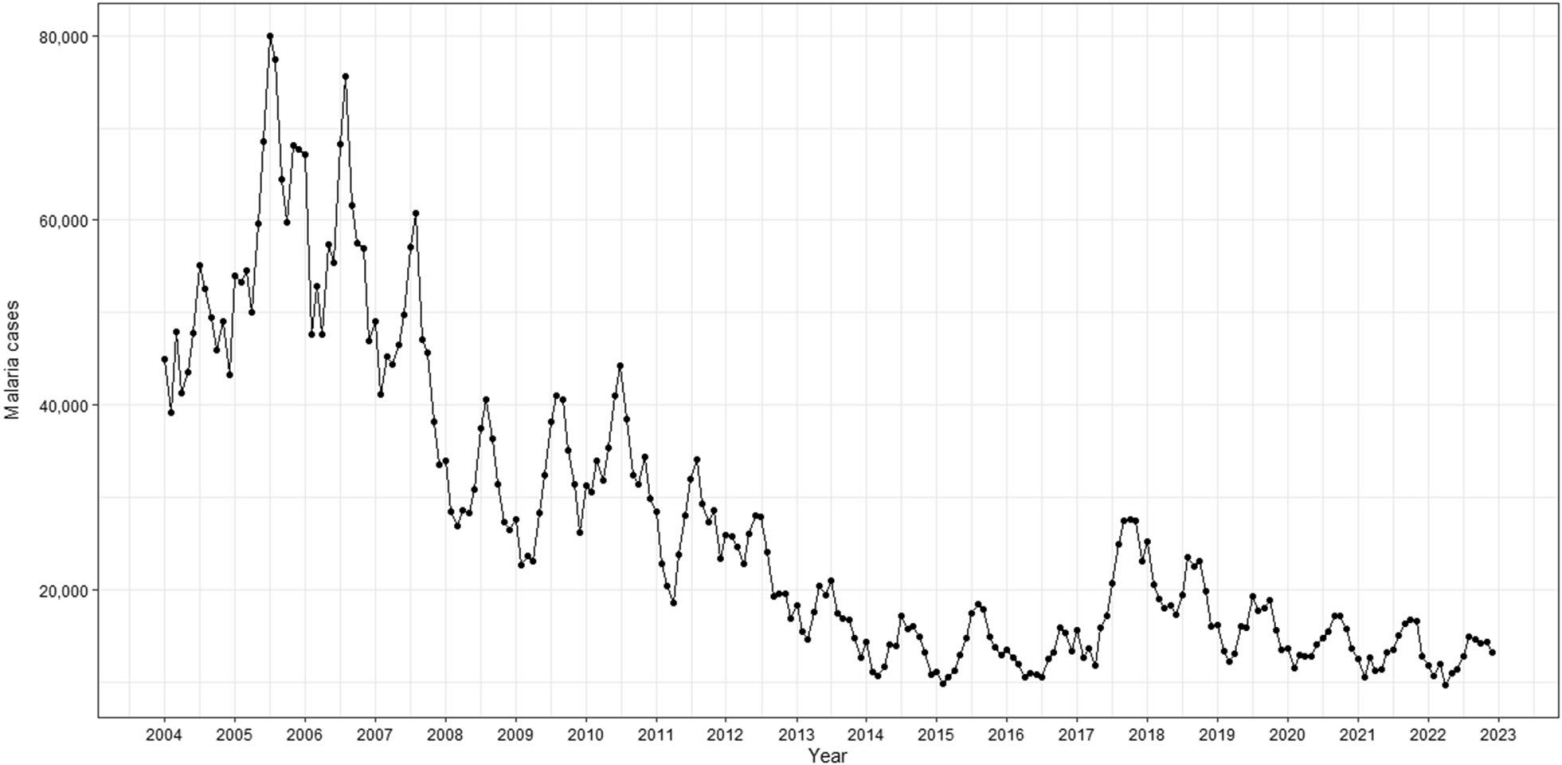
Monthly malaria notifications in the Brazilian Amazon region, 2004- 2022. Source: Malaria epidemiological Surveillance system (Sivep-Malaria)

Between 2004 and 2022, a total of 6,176,878 new cases of malaria were recorded in Brazil. The peak occurred in 2005, with 757,514 notifications, representing a 35.2% increase from 2004. Subsequently, there was an average annual reduction of 7.3% in notifications from 2006 to 2022. In 2022, there were 150,919 registered malaria cases in Brazil (Fig. 3). Notably, during this period, the states with the highest number of notifications were Amazonas (35.3%), Pará (23.5%), and Rondônia (15.0%).

**Fig. 4:**
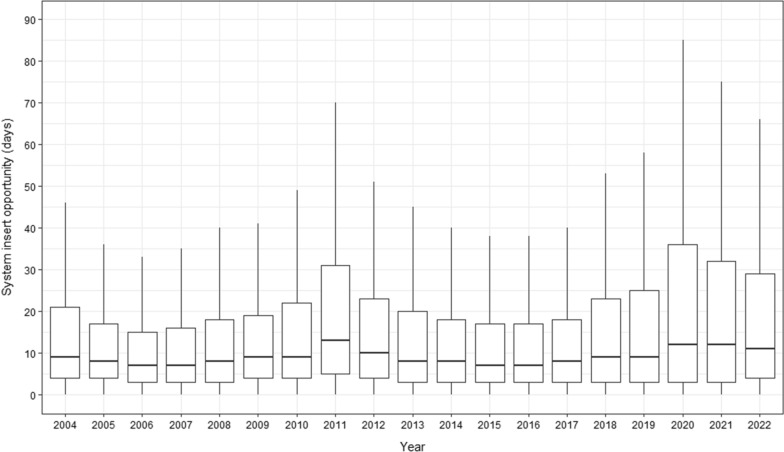
Malaria notification system insert opportunity by year, Brazilian Amazon Region, 2004–2022. *Outliers removed from visualization. Source: Malaria epidemiological Surveillance system (Sivep-Malaria)

The average for data entry opportunity for the entire period was of 17.9 days and a median of 8 days (1st Quartile: 3; 3rd Quartile: 21; Min: 0, Max: 2,213). The best data entry opportunity averages were recorded in 2006, 2007, 2015, and 2016, at 7 days, while the highest averages were in 2011 (13 days on average) and 2020 and 2021 (average of 12 days). Notably, there was a decline in data entry opportunity quality starting from 2016, particularly in 2020 and 2021, possibly due to the effects of the Covid-19 pandemic on malaria epidemiological surveillance activities (Fig. 4).

**Fig. 5:**
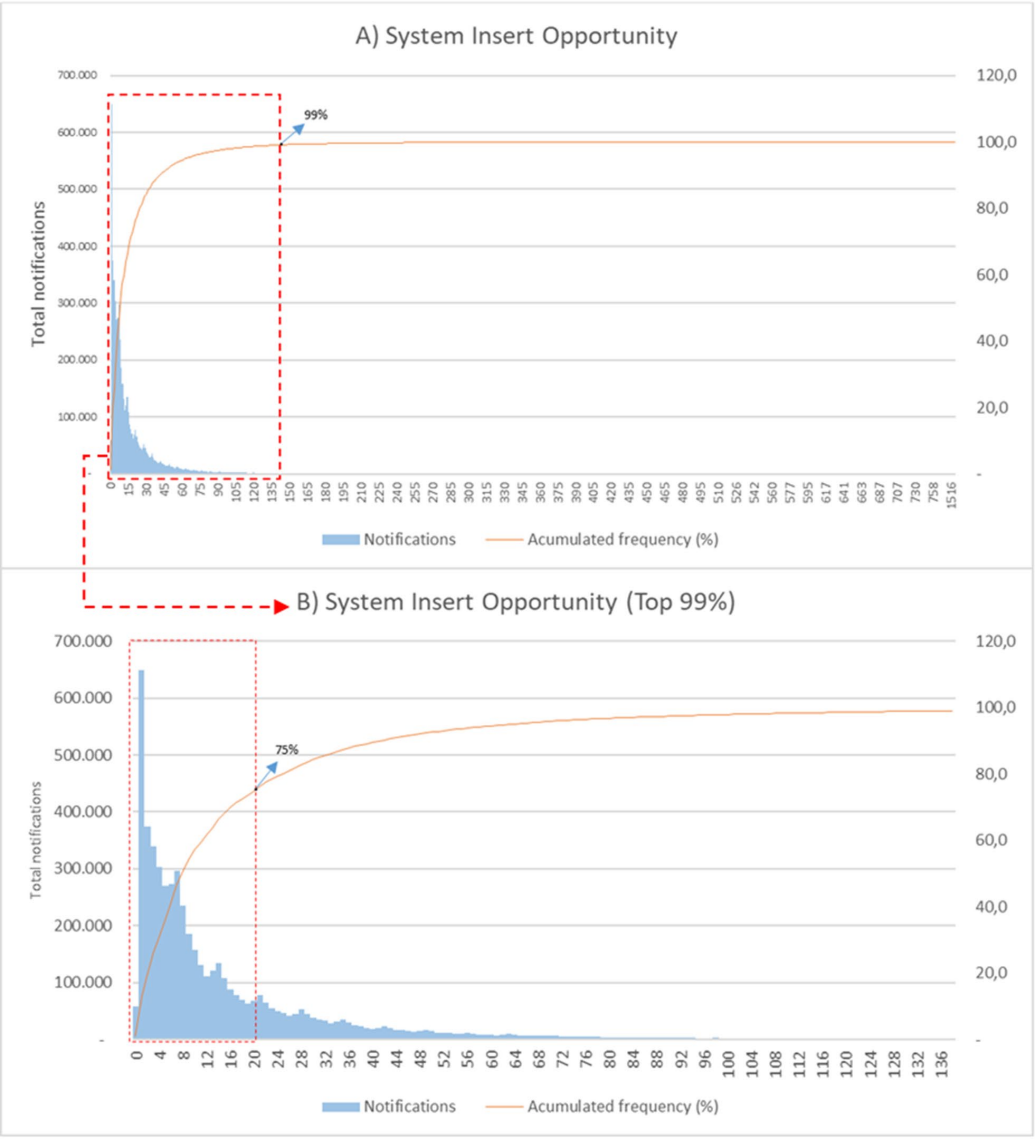
**A** Distribution of malaria notification entry time and cumulative relative frequency, Brazilian Amazon, 2004–2022. **B** Distribution of top 99% malaria notifications. Source: Malaria epidemiological Surveillance system (Sivep-Malaria)

Although the average and median recorded in the period were 17.9 days and a median of 8 days, respectively, the value that most frequently occurs among the notifications is a data entry opportunity of 1 day (Fig. 5A). This value is dispersed, reaching records that took up to 2,213 days to be entered. It can be observed in Fig. 5B that 99% of all notifications were entered within 138 days, with 75.0% being entered within 20 days after notification. Only 19.7% of notifications are entered within 48 h after notification.

**Fig. 6:**
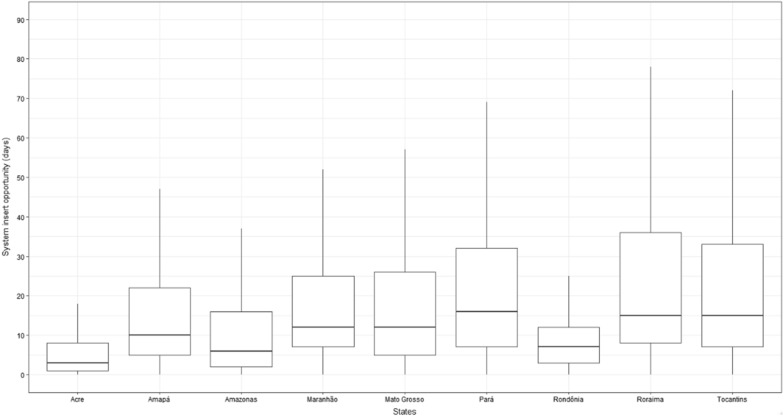
Malaria notification System Insert Opportunity by State, 2004–2022. *Outliers removed from visualization. Source: Malaria epidemiological Surveillance system (Sivep-Malaria)

When analysing this data entry opportunity by state, it was identified that the states with the worst data entry opportunities are Roraima, Tocantins, and Pará, with an average of 31.3, 31.0, and 25.3 days, respectively (medians of 15, 15, and 16 days, respectively). The states with the best opportunities were Acre (average: 10.1; median: 3), Amazonas (average: 15.2; median: 6), and Rondônia (average: 10.2; median: 7) (Fig. 6).

**Fig. 7:**
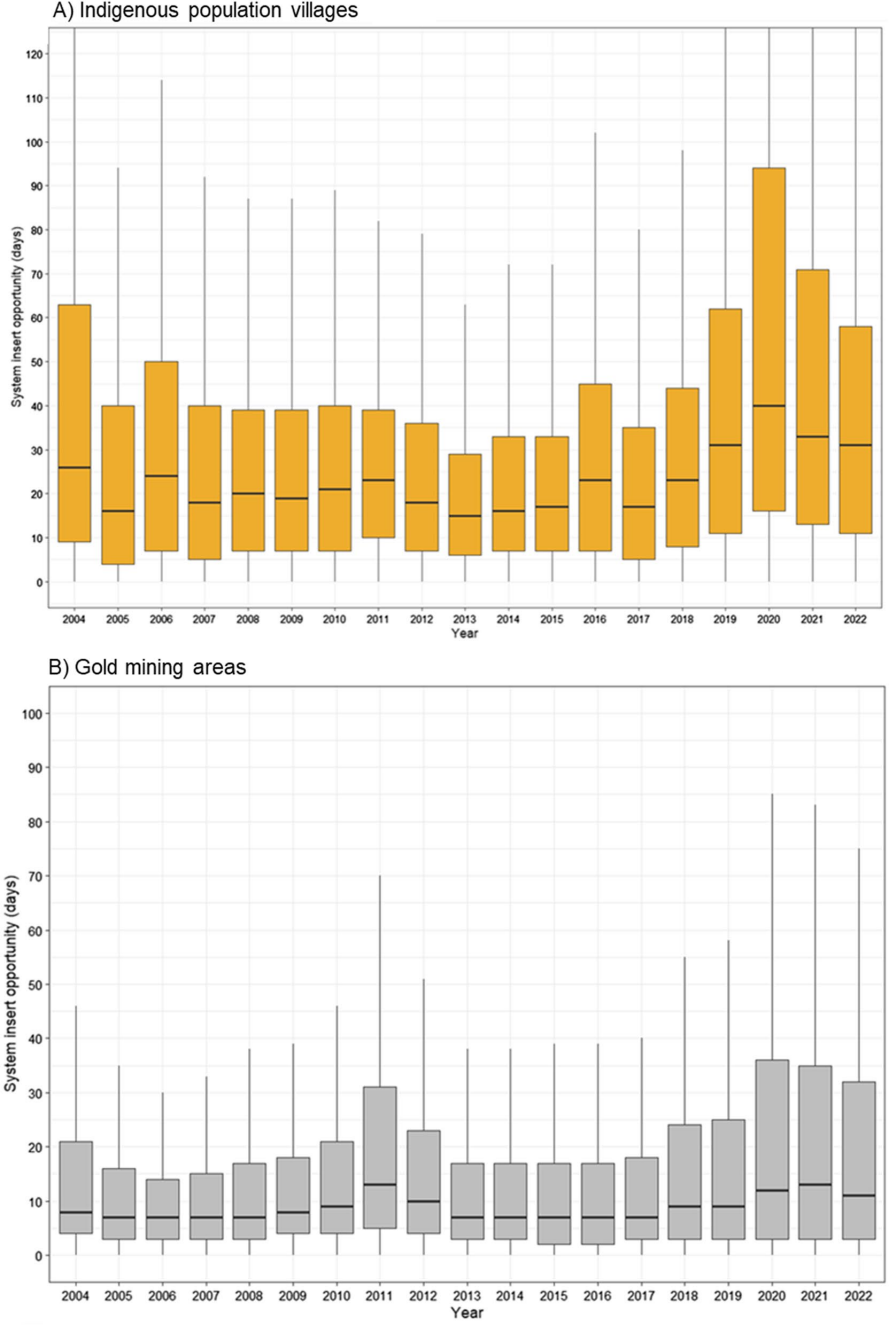
Malaria notification system insert opportunity in cases infected in indigenous population villages (**A**) and gold mining areas (**B**), 2004–2022. *Outliers removed from visualization. Source: Malaria epidemiological Surveillance system (Sivep-Malaria)

Zooming into the indigenous population villages and gold mining areas, the median data entry opportunity is 23 and 15 days, respectively. Specifically for indigenous population villages, the data entry opportunity annual median presented a constant and variation between 2004 and 2018, ranging between 16 and 26 days; after 2018, that opportunity got weaker, reaching over 31 days between the date of notification and the date of data entry (Fig. 7A). In Gold mining areas the data entry opportunity presented a constant median over the lasted 5 years, ranging between 9 and 11 days (Fig. 7B). The average notification times for indigenous villages were 35.7 days and in gold mining areas were 23 days.

## Discussion

### Main results

This study highlighted that the System Insert Opportunity is generally constant and historically stable, with approximately 75% of notifications made within 20 days of the initial report. However, there are variations in the System Insert Opportunity among states, with some states presenting concerning situations. Additionally, when examining notifications in indigenous areas, the variability of the System Insert Opportunity is significant, showing extremely high averages and medians, which is worrisome—especially considering that malaria cases in these areas are expected to increase in the coming years [6].

These results may be linked to the challenges health surveillance actions face in remote areas, particularly due to geographical access difficulties unique to the Amazon region. These can include, for example, the time it can take to return to a health unit after a field visit. Additionally, health teams may experience a shortage of funding for actions and workers available for routine epidemiological surveillance activities and data entry into the system. Often, personnel are tasked with multiple responsibilities, along with bad infrastructure, can delay tasks such as entering notifications into the system [14].

### A mobile module of Sivep-Malaria

Utilizing a mobile application for Sivep-Malaria could enhance the comprehensive notification of malaria cases [15], transmitting them to the national Sivep-Malaria system more promptly, as was seen in a similar strategy in Myanmar—where a mobile phone application implemented in the country improved malaria surveillance with a more opportune and accurate data [16]. This would expedite case notifications and streamline completing the notification form in digital format. The primary users of this application would be healthcare professionals responsible for manually filling out the malaria case notification form in the Amazon region.

Computerizing the notification process can save printing resources and logistics for delivering forms to each case-notifying unit and save physical storage space for forms at MHDs. Moreover, it can reduce rework, as the agent fills out the form manually and the data entry personnel enter the data into the system from the received form.

Indeed, Brazil's malaria epidemiological surveillance system could benefit from technological improvements when considering the change from a control program to an elimination program, similar to what was reported previously in literature [17, 18]. Analysing the data of notifications of new malaria infections in the Amazon region from 2018—the last year Brazil recorded more than 194,000 cases of the disease [6]—the opportunity for transmission of information between the local and national levels would be significantly enhanced, as it can be seen that the notifications in some states, and in particular locations such as indigenous populations villages and in gold mining areas present a long time to have its notification typed into the system.

The national average time for notification is 17.9 days. In comparison, the average notification times for indigenous villages (35.7 days) and gold mining areas (23 days) are longer than the period it takes for patients to produce gametocytes after infection. For *P. vivax* infections, gametocyte production can begin as early as one day after infection, while *P. falciparum* may take up to 7 days after infection. Therefore, that can delay interventions and keep the transmission chains active. Optimizing this opportunity will also allow for timely detection of risk scenarios, such as outbreaks [19, 20].

It is essential that the testing of this application is done in health units outside those with the highest number of case notifications in the Amazon region. As shown by Lana et al*.* [21], more than half of the cases of *P. vivax* and *P. falciparum* are concentrated in specific health units in the Amazon region. Testing such strategy in an environment where cases are less frequent allows for identifying flaws and improving of the computerization process of notifications without jeopardizing routine case reporting. As reported by Ahn et al*.* [19], building and operationalizing an application for case notification involves various methods for capturing, updating, accuracy, integrity, and visualization of data. Considering this, adaptations and adjustments in the constructing the operating system would be necessary until the final product is ready.

This application could also allow acquisition of spatial information through the Global Positioning System (GPS). That would enable the georeferencing of malaria notifications, allowing for a refinement in the health situation analyses at the municipal, state, and federal levels [22].

Therefore, a digital module for mobile devices is suggested to be developed in Sivep-Malaria. This module would enable healthcare professionals to submit case notifications online or offline, particularly beneficial in remote areas such as indigenous population areas, mining sites, and settlements [23, 24]—upon connecting the device to the internet, the notification would be sent automatically to municipality, state, and federal levels. It is important to emphasize that despite the online notification, data backup should be performed on each device and stored in MHDs. Management models for data collection, storage, and transmission that can serve as references are the SIS developed by Ahn et al*.* [9] and the Research Electronic Data Capture—REDCap [25].

A complete, high-quality, and timely notification is crucial for municipal, state, and federal health surveillance managers to formulate strategic actions for disease control. However, what is frequently observed in the field is the omission of information in many fields of the case notification forms, often leading to incomplete notifications [26].

Melo et al*.* [27] reported that the manual or bureaucratic notification system requires a significant amount of time to notify, excessively long or inadequate notification forms, and is one of the factors associated with difficulties in completing notification forms.

Wiefels et al*.* [28] conducted a study to examine the primary sources of errors in the Sivep-Malaria database. They identified that notifications are inconsistent across municipalities and vary across the years studied. The authors further demonstrated that the highest data quality is obtained from information provided by notifying agents. They highlighted that notifications based on patient reports tend to be more inaccurate [28]. Targeting inaccuracy is essential to improve epidemiological surveillance since it has been reported that inaccuracy is a factor that directly influences the performance of Record Linkage processes—a technique that has been widely employed internationally and in the Brazilian territory for studies that utilize information from various HIS that are not integrated [29, 30].

Moreover, with regards to the notification of malaria cases, healthcare professionals may not fully complete case notification forms for various reasons. These reasons may include a lack of commitment on the part of the notifiers, competing priorities among authorities responsible for malaria control at the local level, a failure to recognize the importance of the collected information, and a bureaucratic perception of the filling process, where this action is dissociated from the quality of control [8]. It is worth noting that the use of smartphone applications for disease surveillance has been shown to double the completeness and timeliness of data [31, 32].

Additionally, the implementation of a digital module could and should be accompanied by some restructuring of the notification file. The current version does not allow cases to be followed up, so it is not possible to check if the malaria cases resulted in hospitalization, death, or relapse [29].

Currently, there are some international systems [25] to facilitate electronic data collection, local data storage on mobile devices, review, cleaning, and automated analysis by trained professionals, such as the one for the Global Trachoma Mapping Project (GTMP) [33].

Mobile devices can serve as an effective tool for data capture in epidemiological surveys. In support of the GTMP, the World Health Organization (WHO) introduced the Tropical Data Information System/TD [34], This system aims to provide standardized training to field teams for data capture using Android technology cell phones. These devices are utilized to both collect and transfer data. The Android operating system facilitates the downloading of survey questionnaires, allowing field teams to input relevant data, and the collected data is stored locally until an internet signal is available for transfer and centralized storage.

### Strengths and limitations

This study underscores the need and benefits of employing digital modules for malaria cases notifications in the Amazon region, particularly within the context of disease elimination efforts. A key advantage of these modules would be their ability to geolocate the infection sites and residences of reported cases. This capability could significantly enhance surveillance processes while ensuring care continuity, especially in areas where patients can be of difficult to locate. Such improvements could potentially enhance the quality of data and expedite the transfer time of notifications from local to central levels.

However, the main limitations of this work are related to the use of secondary data from Sivep-Malaria. These data are subject to inaccuracies due to varying investigation and notification procedures across different states. Consequently, the ecological design of this study tends to aggregate information broadly without addressing specific details of individual municipalities.

Despite these challenges, the findings are derived from a robust and well-established system. Therefore, while acknowledging the limitations, there is confidence that the results presented accurately reflect the general and common reality of the Amazon region. Moreover, it is evident that the implementation and development of this digital module must take into account the diverse state and municipal realities. Nevertheless, its most significant benefit would be the enhancement and practice of the concept of health equity.

## Conclusions

In the context of malaria elimination, surveillance should be the primary strategy for assessing each reported case. Reducing notification time, improving data quality and being able to follow-up cases through computerized reports can significantly benefit cases investigation. This can effectively trigger reactive case detection and focal interventions to interrupt local malaria transmission.

While the Sivep-Malaria has been a robust and helpful system for 20 years, it requires ongoing evaluations and improvements. Therefore, the NMCP should continue to enhance its technological infrastructure, re-evaluate training processes, and raise awareness among malaria control centres in municipalities and states. Additionally, there should be an investment in manpower for specialized information technology. The technological improvement of Sivep-Malaria could yield substantial benefits for malaria control in Brazil, assisting the country in achieving disease elimination and fulfilling the Sustainable Development Goals.

### Supplementary Information

**Additional file 1**. Malaria case notification file for the Malaria Epidemiological Surveillance System (Sivep-Malaria).

## Data Availability

All data is available upon reasonable request.

## Abbreviations

HIS: health information systems
SUS: Unified health system
NMCP: National Malaria Control and Prevention Programme
Sivep-Malaria: Malaria epidemiological surveillance information system
MHDs: Municipal Health Departments
GTMP: Global trachoma mapping project
WHO: World Health Organization
