# Supplementary material for: Towards malaria elimination: a reflection about digital notification modules to improve malaria cases notification speed and follow-up in the Brazilian Amazon region
Source: Malar J. 2024 May 23;23:162. doi: 10.1186/s12936-024-04971-6 (PMC11119395; doi:10.1186/s12936-024-04971-6)
Supplement: Supplementary file 1 — Additional file 1. Malaria case notification file for the Malaria Epidemiological Surveillance System (Sivep-Malaria). [file 12936_2024_4971_MOESM1_ESM.docx]

**Additional File I**. Malaria case notification file for the Malaria Epidemiological Surveillance System (Sivep-Malaria).

**
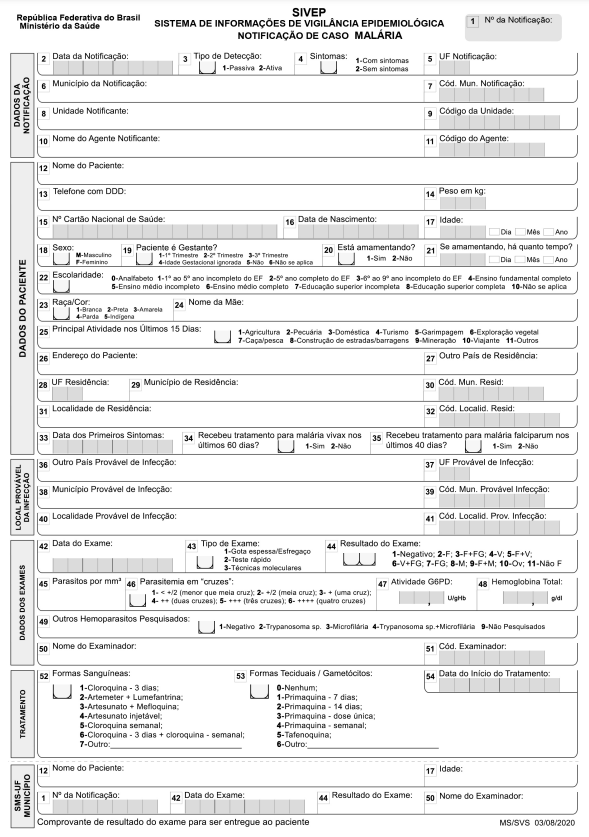
**

Source: Brazilian Ministry of Health. Available at: https://www.gov.br/saude/pt-br/centrais-de-conteudo/publicacoes/svsa/malaria/vigilancia-epidemiologica-da-malaria/ficha-de-notificacao-sivep-malaria-v03-08-2020.pdf/view
